# Influence of White Spot Lesion Severity on the Structural, Optical, and Mechanical Outcomes of Resin Infiltration: An In Vitro Study

**DOI:** 10.3390/diagnostics16070970

**Published:** 2026-03-24

**Authors:** Dan Boariu, Sorana Maria Bucur, Clara Diana Haddad, Elina Teodorescu, Mahmoud El Saafin, Mariana Păcurar

**Affiliations:** 1 Doctoral School, George Emil Palade University of Medicine and Pharmacy, Sciences and Technology, 540142 Targu Mures, Romania; dan_boariu17@yahoo.com; 2 Department of Dentistry, Faculty of Medicine, “Dimitrie Cantemir” University of Târgu Mureș, 3-5 Bodoni Sandor Str., 540545 Targu Mures, Romania; 3 Department of Orthodontics, Faculty of Dentistry, “Grigore T. Popa” University of Medicine and Pharmacy, 700115 Iasi, Romania; 4 Department of Orthodontics and Dentofacial Orthopedics, Faculty of Dental Medicine, Carol Davila University of Medicine and Pharmacy, 050474 Bucharest, Romania; elina.teodorescu@umfcd.ro; 5 Department of Orthodontics, Faculty of Dental Medicine, “George Emil Palade” University of Medicine and Pharmacy, Sciences and Technology, 540139 Targu Mures, Romania; dr_mahmoudsaafin@yahoo.com (M.E.S.); mariana.pacurar@umfst.ro (M.P.)

**Keywords:** white spot lesions, resin infiltration, enamel demineralization, lesion depth, optical masking effect, surface microhardness

## Abstract

**Background and objectives:** White spot lesions (WSLs) represent a common enamel demineralization complication associated with fixed orthodontic treatment. Resin infiltration is widely used as a minimally invasive approach to arrest lesion progression and improve esthetics; however, the influence of lesion severity on treatment effectiveness remains insufficiently understood. This in vitro study aimed to investigate how different severities of white spot lesions influence the structural, optical, and mechanical outcomes of resin infiltration. **Materials and Methods:** Ninety extracted human premolars were subjected to controlled acidic demineralization to produce mild, moderate, and severe lesions. All specimens were treated using a standardized resin infiltration protocol. Lesion depth, resin penetration, optical masking effect (ΔE), and surface microhardness were evaluated using confocal microscopy, spectrophotometry, and Vickers hardness testing. **Results:** Lesion depth increased significantly with demineralization duration (*p* < 0.001). Resin penetration showed a strong positive correlation with lesion depth (r = 0.81), while infiltration efficiency was highest in moderate lesions. Optical masking effectiveness decreased significantly with increasing lesion severity (*p* < 0.01). Surface microhardness improved significantly after infiltration in all groups, with the greatest recovery observed in moderate lesions. **Conclusions:** Lesion severity significantly influences the structural, optical, and mechanical outcomes of resin infiltration. Early and moderately developed WSLs respond more favorably to infiltration treatment, emphasizing the importance of timely intervention during orthodontic therapy. The integrated evaluation of penetration depth, color masking, and microhardness recovery provides a comprehensive understanding of how lesion severity influences the performance of resin infiltration.

## 1. Introduction

White spot lesions (WSLs) are among the most frequent and clinically relevant adverse effects associated with fixed orthodontic treatment, representing the earliest clinically detectable manifestation of enamel demineralization [1]. These lesions are characterized by subsurface mineral loss and appear clinically as opaque, chalky-white areas due to alterations in the refractive index of enamel [1,2]. Their development results from an imbalance between demineralization and remineralization processes at the tooth surface, typically associated with prolonged plaque accumulation and increased activity of cariogenic bacteria around orthodontic appliances. Importantly, the structural characteristics of WSLs vary according to lesion severity and duration of acidic exposure, factors that may directly influence the effectiveness of minimally invasive therapeutic approaches such as resin infiltration [1,2,3,4].

Despite advances in orthodontic materials and techniques, fixed appliances continue to create plaque-retentive niches around brackets, ligatures, and archwires, facilitating the accumulation of acidogenic biofilm and a sustained decrease in local pH [3,4]. Recent studies consistently report a high incidence of WSLs in orthodontic patients, with lesions developing within weeks of appliance placement, particularly in the presence of inadequate oral hygiene [1,2,5].

Clinically, WSLs represent more than an aesthetic concern. Although initially non-cavitated, they reflect structurally weakened enamel with increased porosity, predisposing the tooth to further demineralization and potential progression to cavitated lesions if left untreated [2,6,7]. Their visual impact often becomes more evident after appliance removal, when patients expect improved dental aesthetics, thereby affecting overall treatment satisfaction [7,8,9].

Early detection and risk assessment are therefore essential in orthodontic care [1,10]. Contemporary diagnostic methods, including digital imaging and quantitative light-induced fluorescence (QLF), enable the identification and monitoring of early enamel changes before cavitation occurs [11,12]. Nevertheless, even with preventive strategies such as oral hygiene reinforcement, dietary control, fluoride application, and biomimetic remineralization systems, complete prevention of WSL formation remains challenging, particularly in high-risk patients [13,14].

Consequently, minimally invasive therapeutic approaches aimed at arresting lesion progression and improving aesthetics have gained increasing clinical relevance [1,7,15]. Among these, resin infiltration has emerged as a micro-invasive technique for the management of non-cavitated enamel lesions [15,16]. By penetrating the porous enamel structure, low-viscosity resins occlude diffusion pathways, inhibit lesion progression, and modify optical properties, thereby reducing the visual contrast between sound and demineralized enamel [16,17,18]. Clinical and experimental studies have demonstrated its effectiveness in both arresting lesion progression and improving aesthetic outcomes [1,7,8,13,14,15,17].

In parallel, ongoing research has focused on optimizing infiltrant materials by improving their mechanical properties, reducing water sorption, and incorporating bioactive components such as fluoride-releasing fillers to enhance long-term stability and preventive potential [18,19,20,21].

Despite this growing body of evidence, important uncertainties remain regarding the influence of lesion severity on infiltration outcomes. Most existing studies have evaluated individual parameters—such as penetration depth, color improvement, or microhardness recovery—without simultaneously analyzing their interrelationships within a unified experimental framework. Furthermore, the extent to which increasing lesion severity modifies the structural, optical, and mechanical response of infiltrated enamel remains insufficiently understood [22,23]. This limitation restricts the ability to define optimal clinical indications for resin infiltration based on lesion stage.

Therefore, the present study aimed to investigate the influence of white spot lesion severity on resin infiltration performance through an integrated analysis of lesion depth, penetration behavior, optical masking effect, and surface microhardness recovery. By simultaneously evaluating these parameters and their correlations, this study seeks to provide a more comprehensive understanding of how lesion severity modulates infiltration efficiency and clinical outcomes in orthodontically induced WSLs.

The null hypothesis tested was that lesion severity does not significantly influence resin infiltration outcomes, including penetration depth, optical masking effect, and surface microhardness recovery.

## 2. Materials and Methods

### 2.1. Study Design

This in vitro laboratory study was designed to evaluate the influence of white spot lesion (WSL) severity on resin infiltration performance under controlled experimental conditions simulating orthodontic demineralization. The experimental framework incorporated a multi-parameter assessment, including lesion depth, resin penetration behavior, optical masking effect, and surface microhardness recovery, to analyze both individual outcomes and their interrelationships.

In addition to the immediate effects of infiltration, specimens were subjected to thermocycling to simulate thermal stresses in the oral environment and to evaluate the stability of infiltration outcomes after artificial aging.

Ethical approval was obtained from the Ethics Committee of the George Emil Palade University of Medicine, Pharmacy, Science, and Technology of Târgu Mureș (Approval No. 2709 and Approval Date 27 December 2024).

Specimen collection and preparation were performed between January and February 2025. The artificial demineralization procedures and resin infiltration treatments were conducted from March to April 2025. Laboratory measurements, including confocal microscopy, spectrophotometric analysis, and microhardness testing, were completed between May and June 2025. Statistical analysis and data interpretation were carried out in July 2025.

A priori sample size calculation was performed using G*Power software (Version 3.1, Heinrich-Heine University, Düsseldorf, Germany). Based on previously published in vitro investigations evaluating resin infiltration penetration depth and microhardness recovery in artificial white spot lesions, a large effect size (f = 0.40) was assumed. The calculation indicated that at least 66 specimens were required to detect statistically significant differences among the experimental groups.

With a significance level of α = 0.05 and a statistical power of 80% for one-way ANOVA comparisons among three groups, the minimum required sample size was calculated as 66 specimens.

To compensate for potential specimen loss during preparation, sectioning, or measurement procedures, the sample size was increased by approximately 35%, resulting in a final total of 90 teeth (30 per group).

### 2.2. Operator Calibration and Blinding

Before the experimental procedures, the operator responsible for specimen preparation and measurements underwent a calibration process using 10 pilot specimens in order to standardize measurement techniques and ensure reproducibility. Measurements were repeated after 48 h to evaluate intra-examiner reliability. The intraclass correlation coefficient (ICC) demonstrated excellent agreement (ICC > 0.90).

Randomization of specimens into experimental groups was performed using a computer-generated allocation sequence. Outcome measurements were performed by an examiner blinded to group allocation to minimize measurement bias.

### 2.3. Sample Selection

Ninety extracted human premolars, obtained following orthodontic treatment indications, were included in the study. Teeth presenting cracks, fluorosis, hypoplasia, restorations, or pre-existing carious lesions were excluded.

After extraction, teeth were cleaned of soft tissue remnants and stored in a 0.1% thymol solution at 4 °C until use to prevent dehydration and microbial contamination.

The specimens were randomly allocated into three experimental groups (*n* = 30 per group) using a computer-generated randomization sequence (Random Allocation Software, version 2.0) to minimize allocation bias. Sound enamel measurements obtained before demineralization served as internal control values for each specimen, allowing for comparison between intact enamel, demineralized enamel, and infiltrated enamel conditions.

### 2.4. Creation of Artificial Orthodontic White Spot Lesions

Standardized artificial WSLs were created on the buccal enamel surfaces using a controlled demineralization protocol designed to simulate plaque-induced mineral loss occurring around orthodontic brackets.

Before demineralization, the enamel surfaces were meticulously cleaned with deionized water. To ensure a standardized area of interest, all surfaces were coated with an acid-resistant varnish, leaving a 4 mm diameter circular window of exposed enamel on the buccal surface. The standardized exposure window was verified using a digital caliper to ensure a uniform lesion area among all specimens. This controlled exposure area ensures that demineralization is localized and quantifiable, facilitating consistent comparisons across different severity groups.

**Figure 1:**
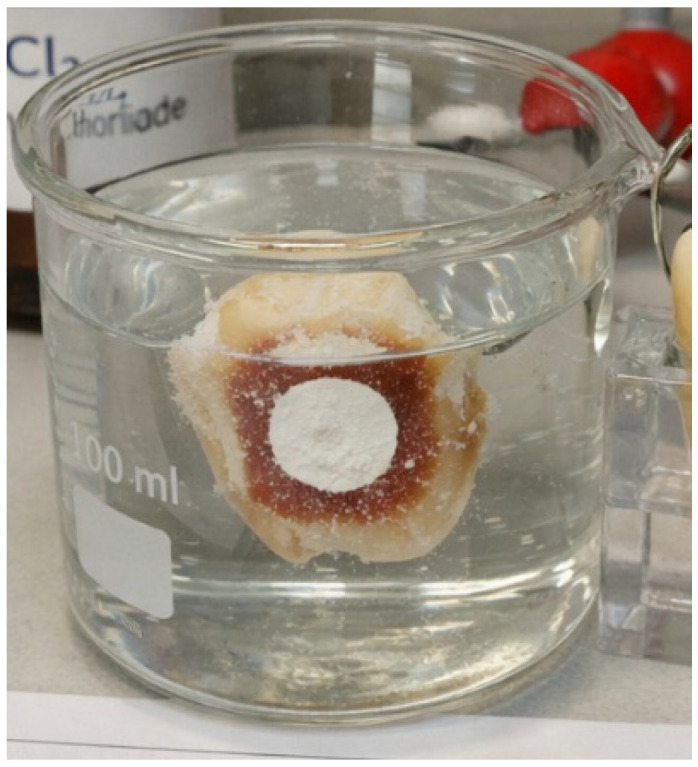
Experimental demineralization.

The specimens were immersed in a demineralizing solution containing lactic acid, calcium chloride, and phosphate (Merck, Darmstadt, Germany), buffered to a pH of 4.5 (Figure 1). This solution simulates the low-pH environment generated by acidogenic biofilm (e.g., *Streptococcus mutans*) during orthodontic treatment. This protocol produces subsurface mineral loss while preserving an intact superficial enamel layer, which is a characteristic feature of clinical white spot lesions [2,5,7,14].

Based on established dental evidence, enamel lesion depth and mineral loss increase predictably with the duration of acidic exposure. Accordingly, three severity groups were defined by immersion time to represent progressive stages of demineralization (Table 1). This protocol reliably produced controlled, reproducible subsurface lesions that closely mimic early clinical demineralization observed during orthodontic treatment. Although artificial lesion models cannot fully replicate the biological complexity of natural white spot lesions, they are widely used in dental materials research because they allow standardized control of lesion depth and mineral loss while reproducing the characteristic subsurface porosity observed in early enamel caries.

The selected demineralization periods (7, 14, and 21 days) were based on previously validated in vitro protocols demonstrating a time-dependent increase in enamel lesion depth and porosity under acidic conditions. Similar exposure durations have been widely used to simulate early, moderate, and advanced stages of enamel demineralization, allowing reproducible differentiation of lesion severity and facilitating comparison with existing literature [6,12,14].

The artificial lesion model used in this study is validated by its high reproducibility, overcoming the biological variability inherent to natural lesions caused by differences in saliva composition and oral hygiene. It represents a reliable surrogate for orthodontic-induced demineralization because it closely mimics the sustained low-pH plaque environment, preserves the characteristic subsurface mineral loss with an intact surface layer, and enables precise standardization of lesion depth, thereby ensuring robust and statistically reliable evaluation of subsequent remineralization outcomes.

### 2.5. Resin Infiltration Procedure

All specimens were treated using a commercially available resin infiltration system (ICON^®^, DMG, Hamburg, Germany) following a standardized protocol.

The procedure consisted of the following steps:

1. Application of 15% hydrochloric acid gel for 120 s;
2. Rinsing for 30 s and air-drying;
3. Application of ethanol-based drying agent for 30 s;
4. Application of infiltrant resin for 3 min;
5. Light curing for 40 s;
6. Second application of infiltrant for 1 min followed by light curing (Bluephase G2, Ivoclar Vivadent, Schaan, Liechtenstein).

All procedures were performed by a single calibrated operator to ensure consistency.

### 2.6. Thermocycling Procedure

Following resin infiltration and initial measurements, all specimens were subjected to thermocycling to simulate thermal fluctuations occurring in the oral cavity during the daily intake of hot and cold beverages.

Thermocycling was performed using a thermocycling device (Thermocycler THE-1100, SD Mechatronik GmbH, Feldkirchen-Westerham, Germany). The specimens underwent 5000 thermal cycles between 5 °C and 55 °C, with a dwell time of 30 s in each bath and a transfer time of 10 s between baths. This protocol is commonly used in dental materials research and corresponds to approximately six months of intraoral thermal stress [14,15]. After completion of thermocycling, color measurements and surface microhardness testing were repeated to evaluate the stability of the optical masking effect and the mechanical reinforcement provided by resin infiltration.

### 2.7. Assessment of Resin Penetration Depth

**Figure 2:**
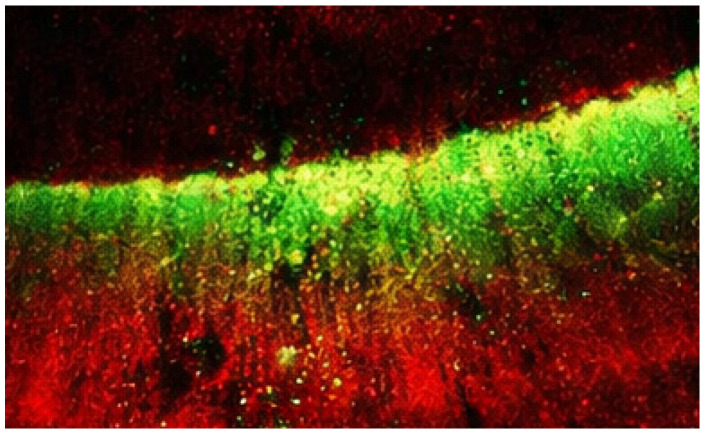
Penetration depth evaluation using confocal laser scanning microscopy. The infiltrant labeled with a fluorescent dye appears as the green signal, indicating the extent of penetration into the lesion body. The red background corresponds to the tooth structure stained with a counterstain, allowing differentiation between infiltrated and non-infiltrated areas.

After infiltration, specimens were sectioned longitudinally through the lesion area. Penetration depth was evaluated using confocal laser scanning microscopy (Leica TCS SP8, Wetzlar, Germany) after staining the infiltrant with a fluorescent dye (Figure 2).

Penetration was measured at three standardized points per specimen, and mean values were calculated. All measurements were performed by an examiner blinded to the experimental group allocation to minimize measurement bias.

### 2.8. Evaluation of the Optical Masking Effect

Color measurements were performed using a Vita Easyshade V spectrophotometer (VITA Zahnfabrik, Bad Säckingen, Germany) based on the CIELab system. Measurements were taken at three timepoints:

- Baseline;
- After WSL creation;
- After resin infiltration.

All color measurements were performed by the same calibrated examiner under standardized lighting conditions, and the operator was blinded to the lesion severity group. Color measurements were repeated after thermocycling to evaluate the stability of the masking effect following artificial aging. Color differences (ΔE) were calculated to determine the masking effectiveness of infiltration across lesion severities.

### 2.9. Surface Microhardness Testing

Surface microhardness was evaluated using a Vickers microhardness tester (HMV-2, Shimadzu, Tokyo, Japan). Indentations were performed on enamel surfaces before infiltration and after treatment. Surface microhardness testing was repeated after thermocycling to assess potential changes in mechanical reinforcement following thermal aging.

The mean of three measurements per specimen was recorded. Before data collection, the examiner performed a calibration procedure using 10 pilot specimens measured twice with a 48 h interval. Intra-examiner reliability was assessed using the intraclass correlation coefficient (ICC), which demonstrated excellent reproducibility (ICC > 0.90).

### 2.10. Statistical Analysis

Statistical analysis was performed using SPSS Statistics Version 26.0 (IBM Corp., Armonk, NY, USA). Data distribution normality was assessed using the Shapiro–Wilk test, and homogeneity of variances was evaluated using Levene’s test.

Descriptive statistics (mean ± standard deviation) were calculated for lesion depth, resin penetration depth, color change (ΔE), and microhardness values. Intra-examiner measurement reliability was evaluated using intraclass correlation coefficients (ICCs).

Comparisons among lesion severity groups were conducted using one-way analysis of variance (ANOVA), followed by Tukey post hoc tests for pairwise comparisons.

Paired *t*-tests were used to evaluate differences between pre- and post-treatment measurements within each group.

Pearson correlation analysis was performed to investigate relationships between lesion depth, penetration depth, optical masking effectiveness, and microhardness recovery.

Comparisons between measurements obtained immediately after infiltration and those recorded after thermocycling were performed using paired *t*-tests.

The level of statistical significance was set at *p* < 0.05. Effect sizes (η^2^) were calculated for ANOVA analyses to estimate the magnitude of differences between lesion severity groups. Additionally, 95% confidence intervals were calculated for the main outcome variables.

## 3. Results

### 3.1. Validation of Artificial White Spot Lesion Formation

Artificial demineralization produced clinically recognizable white spot lesions in all specimens. Confocal microscopy confirmed the presence of subsurface enamel demineralization characterized by an intact superficial layer and increased porosity beneath the surface.

Lesion depth increased significantly with demineralization duration. Mild lesions exhibited shallow subsurface mineral loss, whereas moderate and severe groups showed progressively deeper enamel involvement (Table 2).

Mean lesion depths were approximately as follows:

- 82 ± 15 µm in the mild group;
- 158 ± 21 µm in the moderate group;
- 274 ± 30 µm in the severe group.

One-way ANOVA showed significant differences among groups (*p* < 0.001). Tukey post hoc analysis confirmed statistically significant pairwise differences between all lesion severity levels.

### 3.2. Resin Penetration Depth

Resin infiltration successfully penetrated enamel lesions in all groups. However, penetration depth varied significantly depending on lesion severity (Table 3).

The greatest penetration depth was observed in the moderate lesion group, while significantly lower values were recorded in both the mild and severe groups.

Mean penetration depths were as follows:

- Mild lesions: 68 ± 12 µm;
- Moderate lesions: 132 ± 18 µm;
- Severe lesions: 145 ± 20 µm.

Although penetration depth increased with lesion severity, the penetration ratio (penetration depth relative to lesion depth) was highest in the moderate group.

ANOVA revealed significant differences in penetration ratio between groups (*p* < 0.001). Moderate lesions demonstrated significantly higher penetration efficiency compared to severe lesions.

### 3.3. Optical Masking Effect

Resin infiltration resulted in a significant reduction in color differences (ΔE values) across all lesion severities (Table 4).

Before treatment, severe lesions exhibited the highest ΔE values compared to sound enamel. After infiltration, ΔE values decreased significantly in all groups.

The greatest optical improvement was observed in the mild and moderate lesion groups, while severe lesions showed a lower degree of color recovery.

Mean ΔE reductions were as follows:

- Mild group: 72% reduction;
- Moderate group: 69% reduction;
- Severe group: 52% reduction.

Statistical analysis confirmed significant differences between severity groups (*p* < 0.01).

Paired *t*-tests showed significant ΔE reduction after infiltration in all groups (*p* < 0.001). ANOVA demonstrated significantly lower masking effectiveness in severe lesions (*p* < 0.01).

### 3.4. Surface Microhardness Recovery

Baseline microhardness values decreased significantly after artificial demineralization in all groups, indicating reduced hardness values compared with sound enamel.

Following resin infiltration, microhardness increased significantly compared to post-demineralization values.

The greatest microhardness recovery was observed in moderate lesions.

Mean Vickers hardness values increased (Table 5):

- From 210 ± 18 HV to 285 ± 20 HV in mild lesions;
- From 180 ± 22 HV to 276 ± 24 HV in moderate lesions;
- From 150 ± 25 HV to 240 ± 27 HV in severe lesions.

**Table 5:** Surface microhardness values before and after treatment.

Group	Sound Enamel (HV)	After Demineralization (HV)	After Infiltration (HV)	Percentage Recovery (%)
Mild WSL	318 ± 22	210 ± 18	285 ± 20	76.5
Moderate WSL	320 ± 25	180 ± 22	276 ± 24	78.9
Severe WSL	322 ± 23	150 ± 25	240 ± 27	61.4

Differences between groups were statistically significant (*p* < 0.05).

Paired comparisons showed a significant microhardness increase after infiltration (*p* < 0.001). ANOVA indicated significantly lower recovery in severe lesions (*p* < 0.05).

### 3.5. Correlation Analysis (Table 6)

**Figure 3:**
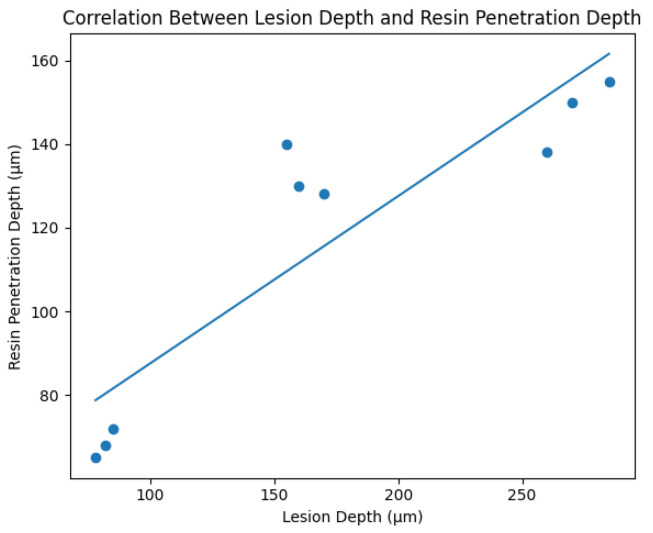
Scatter plot illustrating the correlation between lesion depth and resin penetration depth in artificial orthodontic white spot lesions. A strong positive linear relationship was observed (R^2^ = 0.806, *p* < 0.001), indicating that increased enamel porosity associated with deeper lesions facilitates greater resin infiltration.

A significant positive correlation was observed between lesion depth and resin penetration depth (r = 0.81, *p* < 0.001). As illustrated in Figure 3, deeper lesions were associated with greater resin infiltration, demonstrating a strong positive linear relationship (R^2^ = 0.806, *p* < 0.001). This finding suggests that increased enamel porosity in deeper lesions facilitates improved resin penetration.

**Table 6:** Pearson correlation analysis between study variables.

Variables Compared	Correlation Coefficient (r)	*p*-Value
Lesion depth vs. penetration depth	0.81	<0.001
Lesion depth vs. optical masking	−0.67	0.002
Penetration depth vs. microhardness recovery	0.58	0.011
Penetration ratio vs. masking effect	0.62	0.006

However, a negative correlation was identified between lesion severity and optical masking effectiveness (r = −0.67, *p* < 0.01). Microhardness recovery showed a moderate positive correlation with penetration depth (r = 0.58, *p* < 0.05).

Thermocycling produced minor reductions in the optical masking effect and surface microhardness values across all groups; however, the overall improvements obtained after resin infiltration remained statistically significant compared with post-demineralization measurements.

No significant differences in the stability of infiltration outcomes were observed between mild and moderate lesions, whereas severe lesions showed slightly greater reductions after thermal aging.

## 4. Discussion

White spot lesions (WSLs) remain one of the most common iatrogenic complications of fixed orthodontic therapy, resulting from persistent biofilm activity and enamel demineralization [1,2,5,7,8,10,12,13,15,17,20,24].

A key strength of the present study is the integrated evaluation of structural, optical, and mechanical outcomes of resin infiltration across different stages of lesion severity. Unlike previous studies focusing on isolated parameters, this approach allows for a more comprehensive understanding of infiltration dynamics, emphasizing that treatment effectiveness depends not only on penetration depth but also on lesion morphology and the preservation of enamel microarchitecture.

Artificial WSL models are widely used due to their reproducibility and ability to standardize lesion depth [25]. In the present study, the demineralization protocol successfully reproduced subsurface lesions with an intact superficial layer, consistent with early enamel caries [26,27]. However, such models cannot fully replicate the biological complexity of natural lesions, where salivary composition, pellicle formation, biofilm activity, and dynamic demineralization–remineralization cycles influence lesion structure and permeability. Therefore, caution is required when extrapolating these findings to clinical conditions.

Thermocycling further demonstrated that the improvements in optical masking and microhardness remained largely stable after artificial aging, suggesting satisfactory short-term durability of the infiltrated enamel structure despite potential thermal stresses at the resin–enamel interface [14,15,27].

The progressive increase in lesion depth observed in this study confirms the time-dependent nature of enamel demineralization under acidic conditions, consistent with previous experimental models simulating orthodontic plaque accumulation [25]. Clinically, this reflects the sustained low-pH environment around orthodontic appliances, where plaque stagnation promotes continuous mineral loss [28].

A strong positive correlation between lesion depth and resin penetration depth (R^2^ = 0.806) confirms the fundamental role of enamel porosity in infiltration behavior [29]. However, when analyzed relative to lesion depth, moderate lesions demonstrated the highest infiltration efficiency, whereas severe lesions showed significantly reduced penetration ratios.

This finding can be explained by microstructural changes during lesion progression. In moderately developed lesions, enamel prism organization is partially preserved, and the porous network remains sufficiently interconnected to support capillary diffusion of the infiltrant [30]. This structure facilitates effective penetration while maintaining a mineral scaffold for polymer stabilization.

In contrast, advanced lesions exhibit extensive mineral loss, disruption of prism architecture, and reduced pore connectivity in deeper regions. These alterations may create diffusion barriers, limit capillary flow, and hinder uniform resin distribution. Additionally, increased diffusion distance and viscosity-related limitations may further restrict complete infiltration, explaining the lower penetration efficiency observed in severe lesions [30,31]. Structural disorganization in advanced lesions may also increase polymerization shrinkage stresses and compromise long-term stability [31].

These findings reinforce previous evidence that infiltration success is strongly dependent on lesion morphology and that early and moderate lesions represent the most favorable therapeutic targets [11,27,32].

Resin infiltration significantly reduced ΔE values across all groups, confirming its effectiveness in improving enamel aesthetics. This effect is attributed to the replacement of air-filled pores with resin, reducing light scattering and restoring refractive index compatibility with sound enamel [33]. However, optical improvement decreased with increasing lesion severity [34]. In severe lesions, incomplete penetration leaves residual porous areas capable of scattering light, resulting in persistent opacity [35]. These results align with clinical studies indicating that optimal aesthetic outcomes are achieved in early-stage lesions, whereas advanced lesions may require combined treatment approaches [2,5,12,32].

Surface microhardness results further support the reinforcing effect of resin infiltration. All groups showed significant improvement after treatment, reflecting the ability of infiltrants to stabilize demineralized enamel. Notably, moderate lesions exhibited the greatest recovery, consistent with their higher infiltration efficiency. In contrast, severe lesions showed limited mechanical recovery relative to lesion depth, likely due to irreversible structural damage that cannot be fully compensated by resin infiltration.

Correlation analysis highlights the multifactorial nature of infiltration outcomes. While lesion depth positively influences penetration, it is negatively associated with optical and mechanical performance. These findings indicate that treatment success depends not only on lesion size but also on the preservation of enamel microarchitecture, which appears critical for both functional and aesthetic outcomes [2,3,13,20,30].

From a clinical perspective, these results emphasize the importance of early detection and timely intervention. Resin infiltration appears most effective when applied during early or intermediate stages of lesion development, where sufficient structural integrity allows optimal penetration and reinforcement. In contrast, advanced lesions may require combined or alternative therapeutic approaches due to reduced infiltration efficiency and limited aesthetic recovery [5,12,14,30].

It should also be noted that enamel alterations following orthodontic treatment are influenced not only by mechanical procedures but also by pre-existing demineralization. WSLs present at the time of bracket removal may already compromise enamel structure, potentially affecting the outcomes of finishing procedures. Previous studies have shown that such lesions can influence enamel loss during cleanup, highlighting the need to consider pre-existing demineralization when evaluating post-orthodontic enamel integrity [36].

From a clinical decision-making perspective, lesion severity should be considered a key determinant when selecting resin infiltration. Moderate lesions appear to represent the optimal therapeutic window, combining sufficient porosity for penetration with preserved structure for effective reinforcement, whereas advanced lesions may require adjunctive strategies.

### 4.1. Clinical Relevance

The findings highlight the importance of early detection and minimally invasive management of WSLs during orthodontic treatment. Resin infiltration is most effective in lesions with limited depth, supporting its use as an early intervention strategy to optimize both aesthetic and mechanical outcomes.

### 4.2. Study Strengths

This study benefits from a standardized and reproducible artificial lesion model, combined with an integrated evaluation of structural, optical, and mechanical parameters. Randomization, examiner calibration, and blinded measurements further enhance the reliability of the results.

### 4.3. Study Limitations and Future Perspectives

The in vitro design does not replicate the full biological complexity of the oral environment, including salivary dynamics, biofilm behavior, and natural remineralization processes. Although thermocycling short-term aging, additional factors such as pH cycling and mechanical loading were not included [37].

Moreover, natural lesions exhibit greater heterogeneity, which may influence resin infiltration differently [3,5,13,17,30]. Future research should include long-term clinical studies and advanced aging models to better evaluate durability. The development of bioactive infiltrants and combined therapeutic approaches may be particularly relevant for managing advanced lesions.

## 5. Conclusions

Lesion severity plays a decisive role in determining the structural, optical, and mechanical outcomes of resin infiltration in white spot lesions. While penetration depth increased with lesion severity, infiltration efficiency was greatest in moderate lesions, indicating that effective resin diffusion depends not only on lesion depth but also on the preservation of enamel microarchitecture.

Increasing lesion severity was associated with reduced optical masking and diminished microhardness recovery, reflecting the limitations of resin infiltration in structurally compromised enamel. These findings identify early and moderately developed lesions as the optimal targets for this micro-invasive approach.

From a clinical perspective, the results define a clear therapeutic window in which resin infiltration provides maximal benefit, emphasizing the importance of early detection and timely intervention during orthodontic treatment. In advanced lesions, reduced infiltration efficiency and limited aesthetic recovery suggest the need for adjunctive or alternative strategies.

These findings contribute to a more precise, evidence-based understanding of resin infiltration, supporting improved clinical decision-making in the minimally invasive management of orthodontically induced white spot lesions.

## Figures and Tables

**Table 1:** Severity groups based on immersion time.

Severity Group	Exposure Duration	Estimated Lesion Depth	Clinical Correlation
Mild	7 Days	~50–100 µm	Early subsurface demineralization
Moderate	14 Days	~120–200 µm	Established WSL with visible opacity
Severe	21 Days	>250 µm	Advanced lesion with high porosity

**Table 2:** Lesion depth according to demineralization duration.

Group	Demineralization Duration	Mean Lesion Depth (µm)	Standard Deviation	Minimum	Maximum
Mild WSL	7 days	82.4	14.7	58	109
Moderate WSL	14 days	158.3	21.2	121	197
Severe WSL	21 days	274.6	29.5	225	331

**Table 3:** Resin penetration depth and penetration efficiency.

Group	Lesion Depth (µm)	Penetration Depth (µm)	Penetration Ratio (%)	Standard Deviation
Mild WSL	82.4	68.1	82.6	12.4
Moderate WSL	158.3	132.7	83.8	17.6
Severe WSL	274.6	145.3	52.9	19.2

**Table 4:** Color changes (ΔE) before and after resin infiltration.

Group	Baseline ΔE	Post-Demineralization ΔE	Post-Infiltration ΔE	Percentage Reduction (%)
Mild WSL	2.3 ± 0.7	14.6 ± 2.4	4.1 ± 1.3	71.9
Moderate WSL	2.5 ± 0.8	16.2 ± 2.9	5.0 ± 1.5	69.1
Severe WSL	2.4 ± 0.6	19.8 ± 3.2	9.5 ± 2.6	52.0

## Data Availability

The datasets generated and analyzed during the current study are available from the corresponding author upon reasonable request.
